# *Leishmania donovani* induced Unfolded Protein Response delays host cell apoptosis in PERK dependent manner

**DOI:** 10.1371/journal.pntd.0006646

**Published:** 2018-07-23

**Authors:** Kumar Abhishek, Sushmita Das, Ashish Kumar, Ajay Kumar, Vinod Kumar, Savita Saini, Abhishek Mandal, Sudha Verma, Manjay Kumar, Pradeep Das

**Affiliations:** 1 Division of Molecular Biology, Rajendra Memorial Research Institute of Medical Sciences, Agamkuan, Patna, Bihar, India; 2 Department of Microbiology, All India Institute of Medical Sciences, Phulwarisharif, Patna Bihar, India; 3 National Institute of Pharmaceutical Education and Research, Export Promotion Industrial Park, Hajipur, Vaishali, Bihar, India; Instituto de Ciências Biológicas, Universidade Federal de Minas Gerais, BRAZIL

## Abstract

**Background:**

Endoplasmic reticulum (ER) stress generated unfolded stress response (UPR) is a basic survival mechanism which protects cell under unfavourable conditions. Leishmania parasite modulates host macrophages in various ways to ensure its survival. Modulation of PI3K-Akt pathway in delayed apoptotic induction of host; enables parasite to stabilize the infection for further propagation.

**Methodology:**

Infected RAW macrophages were exposed to campothecin or thagsigargin and phosphorylation status of PERK, Akt, BAD and Cyt-C was determined through western blotting using phospho specific antibody. Expression at transcriptional level for cIAP1 &2, ATF4, CHOP, ATF3, HO-1 and sXBP1 was determined using real time PCR. For inhibition studies, RAW macrophages were pre-treated with PERK inhibitor GSK2606414 before infection.

**Findings:**

Our studies in RAW macrophages showed that induction of host UPR against *L*.*donovani* infection activates Akt mediated pathway which delays apoptotic induction of the host. Moreover, Leishmania infection results in phosphorylation and activation of host PERK enzyme and increased transcription of genes of inhibitor of apoptosis gene family (cIAP) mRNA. In our inhibition studies, we found that inhibition of infection induced PERK phosphorylation under apoptotic inducers reduces the Akt phosphorylation and fails to activate further downstream molecules involved in protection against apoptosis. Also, inhibition of PERK phosphorylation under oxidative exposure leads to increased Nitric Oxide production. Simultaneously, decreased transcription of cIAP mRNA upon PERK phosphorylation fates the host cell towards apoptosis hence decreased infection rate.

**Conclusion:**

Overall the findings from the study suggests that Leishmania modulated host UPR and PERK phosphorylation delays apoptotic induction in host macrophage, hence supports parasite invasion at early stages of infection.

## Author summary

Visceral Leishmaniasis or Kala-azar is one of the severe tropical neglected parasitic diseases caused by *Leishmania donovani* in Indian subcontinent. Modulation of host in terms of delayed apoptotic induction is one of the aspects which favours disease establishment; however the mechanism is not clearly understood yet. In the present study, we tried to explore the connection between *L*.*donovani* infection induced UPR in host with delayed onset of apoptosis. We found that *L*.*donovani* infection phosphorylates the PERK and Akt molecule in host along with delayed apoptosis. Simultaneously, the levels of cellular IAP (cIAP1 & 2) genes were also up-regulated in infected macrophages. To assess the involvement of PERK in delayed apoptosis of host, we inhibited the phosphorylation of PERK under the exposure to apoptotic inducers. We found that PERK inhibition decreased the Akt phosphorylation and fails to activate other associated downstream molecules involved in delayed apoptosis of host. Also, a significant reduction in cIAP levels was observed. Under oxidative exposure, inhibition of PERK phosphorylation debilitates infected RAW cell’s ability to maintain redox homeostasis leading to higher nitric oxide production. Altogether, *L*.*donovani* infection modulates host apoptosis in a PERK dependent manner and favours infection.

## Introduction

*Leishmania* ability to defy the host immune response is a major cause for persistence of Leishmaniasis. Parasite modulates host in various aspects and hampers the activation of adaptive immune responses against infection [1]. Expression of LPG on parasite surface and TLR 2 modulates the host immune response [2, 3] by providing resistance to complement, attachment and entry into macrophages, protection against proteolytic damage within acidic vacuoles [4] or inhibition of phagosomal maturation [5]. *Leishmania* parasite has to counter the oxidative and nitrosative pressure generated in host macrophage against invasion. Parasite modulates host NO and IL-12 production [6, 7, 8, 9] and induces host HO-1which suppress the production of superoxide and increases parasitic burden [10].

Unfolded protein response (UPR) is an evolutionary conserved mechanism that restores cellular homeostasis and ensures cell survival during Endoplasmic Reticulum stress. UPR consists of 3 signalling pathways: activation of transcription factor (ATF)-6, inositol-requiring enzyme (IRE)-1, and PKR-like endoplasmic reticulum kinase (PERK) [11]. As a downstream consequence, activated IRE1 increases cytosolic concentration of spliced XBP1 (sXBP1); an active transcription factor which in turn activates an array of genes to counter ER stress for cell survival [12, 13, 14]. *L*. *amazonensis* induces the IRE1a/XBP1 pathway to escape cellular defence; particularly oxidative stress by sXBP1 induced expression of antioxidant molecules such as SOD-1 and catalase, and increases expression of IFN1-β, an important cytokine that favour’s *L*. *amazonensis* infection [15].

In another aspect of modulation, *Leishmania* parasite induces a mild ER stress in host macrophages and activates the PI3K-Akt pathway [16]. However, a very few studies has been conducted in correlation with induced UPR and leishmaniasis, but the PI3K-Akt phosphorylation is of great importance in parasite biology. As a part of host modulation, *Leishmania* promastigote activates host PI3K-Akt pathway, phosphorylates BAD, checks cytochrome-C leakage from mitochondria, inhibits caspase activation; hence delays apoptotic induction in host macrophage [17].

PERK being an important component of integrated endoplasmic reticulum stress response (IERSR) [18]; its activation influences cellular adaptation through multiple mechanisms [19, 20]. Induction of mammalian inhibitor of apoptosis proteins (IAP proteins) is one of the PERK-dependent survival mechanisms which protects cell from ER stress- induced apoptosis [21, 22]. Also, activation of the PI3K–Akt pathway by ER stress is dependent on PERK [23]. Also, PERK phosphorylation under oxidative stress is crucial for protection against oxidative stress and maintaining redox balance for cell survival [24, 25].

So, in this study we first confirms the previous findings that *Leishmania* infection delays host cell apoptosis under exposure to apoptotic inducers by phosphorylating Akt, BAD protein which inhibits the caspase-3 activity, resulting delayed apoptosis. Further we investigated the effect of *L*.*donovani* infection in imparting endoplasmic reticulum (ER) stress in host and activation of unfolded protein response (UPR). Here, we first time reported that *Leishmania* infection induced host UPR relays on PERK activation. Our findings suggest that inhibition of PERK phosphorylation render RAW cells more prone to apoptosis ultimately leading to decreased infection rate. Moreover, activation of IAP (inhibitor of apoptosis proteins) class of proteins which protects cell from various apoptotic stimulus; depends upon PERK phosphorylation. Overall we conclude that UPR activated against *L*.*donovani* infection is a PERK dependent process and have an important role in delayed onset of apoptosis in host macrophages; hence contributes to *Leishmania* infectivity.

## Methods

### Leishmania cell culture

*Leishmania donovani* promastigotes used in all experiments were of clones AG83 (MHOM/IN/ 1983/AG83). The promastigotes were grown in fresh M199 media (Gibco) in 25 cm^2^ flasks (Nunc) at 25°C supplemented with 10% FBS (Gibco). Cultures were allowed to reach stationary phase (5–6 days post inoculation), as determined by growth curve analysis (S1 Fig), prior to inoculation into fresh medium. Syrian golden hamsters were used to maintain the infectivity of the clone regularly [26].

### Cell line maintenance

The RAW 267.4 cell line (obtained from NCCS, Pune) was maintained in a 25-cm2 flask in RPMI-1640 medium (Gibco) supplemented with 10% FBS and antibiotics (streptomycin and penicillin) in a humidified 5% CO_2_ incubator (Sanyo, Japan) at 37°C. The growth and condition of cultures were routinely checked under an inverted microscope (Nikon, USA). The cells were sub cultured in every 72 hrs.

### Real-time PCR

RAW267.4 macrophages were allowed to infect with *L*.*donovani* promastigotes for 4 h, washed to remove unattached promastigotes and further infection was carried out for mentioned time periods (4 h /12 h/ 36 h) before campothecin (2mM/ 6 h) or thapsigargin (1μM/ 1hr) treatment. For inhibition studies, RAW267.4 macrophages were pre-treated with GSK2606414 for 2 hours before allowing infection. Reverse transcription was performed using 0.2 mg total RNA using an anchored oligo (dT) (H-dT11M, where M represents A, C, or G; Gen- Hunter). Real-time PCR, was performed in the Light Cycler 480 (Roche) using SYBR green (Roche) chemistry. The cycling conditions were; 1 cycle at 95°C for 3 min and 40 cycles of 95°C for 15s (denaturation), 58°C for 30s (annealing), and 72°C for 30s (extension). The fluorescence signal was captured at the end of each cycle using the SYBR channel (excitation wavelength 490-nm and emission wavelength 525-nm). Results expressed as target/reference ratios of each sample, normalized by the target/reference ratio of the calibrator. Here, the target/reference value of untreated/normal RAW macrophages was used as the calibrator and the GAPDH gene was used internal control/ reference to normalize the qRT-PCR in the experiments. The primers used for RT-PCR are mentioned in tabular form. (Table 1).

**Table 1:** Primer sequences for real-time PCR.

Gene	Forward primer (5'-3')	Reverse primer (5'-3')
*ATF3*	GCCATCCAGAACAAGCACCT	GGCTACCTCGGCTTTTGTGAT
*ATF4*	CCCTTCACCTTCTTACAACCTC	TGAAGGAGATAGGAAGCCAGAC
*sXBP1*	CTGAGTCCGCAGCAGGT	TGTCCAGAATGCCCAACAGG
*CHOP*	GGAGCATCAGTCCCCCACTT	TGTGGGATTGAGGGTCACATC
*cIAP1*	GAAGAAAATGCTGACCCTACAGA	GCTCATCATGACGACATCTTTC
*cIAP2*	CGATGCAGAAGACGAGATGA	TTTGTTCTTCCGGATTAGTGC
*HO-1*	GCAGAGAATGCTGAGTTCATG	CCTCCTCCAGGGCCACATAGATGTG

### Western blotting

RAW 264.7 cells were plated in 6-well polystyrene plates 1 d before infection and were either pre-treated with GSK2606414 or untreated. Total protein extracts were prepared from infected cells 4 h, 12 h or 36 h after infection. For preparation of total cellular extracts, macrophages were washed 3 times with PBS and then lysed in 100 ml lyses buffer [50mMTris- HCl (pH 7.5), 5 mM EDTA, 10 mM EGTA, 50 mM NaF, 20 mM b-glycerophosphate, 250mM NaCl, 0.1%Triton X-100, and 1mg/ml bovine serum albumin) supplemented with 1:100 dilution of protease inhibitor cocktail (Sigma-Aldrich). Total protein (50μg) was separated by SDS-PAGE. The proteins were transferred to PVDF membranes (Bio-Rad, Hercules, CA, USA) and blotted with antibodies against phosphos Akt (Santa Cruz Biotechnology, Dallas, TX, USA), phosphos PERK (Cell Signaling), phosphos BAD (Santa Cruz Biotechnology), GAPDH (Santa Cruz Biotechnology), anti-PERK (Cell Signaling) overnight. Membranes were incubated with horseradish peroxidase–conjugated IgG (1:5000) for 1 h in room temperature and washed extensively with Tris-buffered saline- Tween (TBST). Antibody antigen complexes were detected by enhanced chemiluminescence kit (Thermo Fischer Scientific, USA) was used for detection. The intensity of bands was analyzed using Quantity One software (Bio-Rad).

### Caspase-3 activity assay

RAW 267.4 macrophages either untreated or pre-treated with GSK2606414 were infected with *L*. *donovani* promastigotes. After desired time periods, the cells were induced with campothecin (2μM/ 6 h) in six well plates (1x10^6^ cells). The cells were then scraped and harvested, lysed in cell lysis buffer supplied with the caspase-3 Fluorometric Assay Kit (BioVision) and the concentration of protein in lysates was determined. Equal amount of protein from each set was used to determine caspase-3 activity. The detection was based on the cleavage of AFC (7-amino-4-trifluoromethyl coumarin) from DEVD-AFC by caspase-3 and the relative fluorescence was measured in the PerkinElmer LS-55 spectrometer (Perkin Elmer, USA) at excitations of 390 to 400 nm and emissions of 510 to 550 nm.

### Detection of DNA fragmentation

Raw cells (1×10^6^) were allowed to adhere into 6- well (Corning, USA) and incubated at 37°C in 5% CO_2_ incubator (Sanyo, Japan)_._ Once macrophages were adhered, pre-treatment with GSK2606414 was given and washed. Further infection was carried out by adding *L*.*donovani* promastigotes to each wells and maintained at 37°C in 5% CO_2_ overnight. Non- internalized promastigotes were removed by gentle washing twice with PBS. Infected macrophages were treated campothecin (6 h/ 2μM) or thapsigargin (1.0μM / 1hr) in 5% CO_2_ at 37°C. Untreated infected macrophages were taken as a control. Total genomic DNA was isolated using an apoptotic DNA laddering kit (Roche, USA) as described earlier [27]

### Inhibition assay

GSK2606414 (Calbiochem), a PERK inhibitor, was added at a 30nM final concentration to RAW 267.4 macrophages, 2 h prior to the infection, washed and the macrophages were allowed to infect with *L*.*donovani* promastigotes (MOI 10:1). Further treatment of campothecin (2μM/ 6 h) [17] or thapsigargin (1.0μM / 1hr) [15] was given.

### Infection study

For infection studies, RAW 264.7 cells were plated in 6-well polystyrene plates 1 day before infection. The infection index was calculated by multiplying the percentage of infected macrophages by the average number of parasites per macrophage on Giemsa-stained slides [15].

### Apoptotic detection

Untreated or GSK2606414 pre-treated RAW 267.4 macrophages were plated in six well tissue culture plates overnight and then infected with late stationary phase *Leishmania* promastigotes. After 4 h, free parasites were washed off with PBS and the treatment of either 2mM campothecin for 6 h or thapsigargin (1μM/ 1hr) was given. Cells were trypsinized and resuspended in phosphate-buffered saline. Propidium iodide was added to a concentration of 2.5 mg/ml [20]. Propidium iodide fluorescence was measured with a Becton Dickinson FACS ARIA.

### Determination of nitric oxide production

Nitric oxide (NO) concentration was determined by analysing nitrite content in cell supernatant with the Griess reaction. *L*.*donovani* infected RAW cells either GSK 2606414 pre-treated or untreated were exposed to H_2_O_2_ (200μM/ 4hr) and Culture supernatant from each set (50μL) was mixed with 50μL of a solution containing sulphanilamide (10 mg/mL), N-[naphthyl] ethylenediamine dihydrochloride (NEED; 1 mg/mL) and 5% phosphoric acid. The absorbance was measured by spectrophotometry at 540 nm [28].

### Statistical analysis

Graph Pad Prism (Version 6.0, GraphPad Software, USA) was used to analyse the data statistically. Each experiment was repeated three times in separate sets and the results were expressed as Mean ± SD. Statistical differences were determined using Mann–Whitney *U* test for comparing two groups or Kruskal–Wallis with Dunn’s multiple comparison test for comparing three or more groups. A *P-* value of <0.05 was considered significant.

## Results

### *L. donovani* infection induces UPR in RAW macrophages

**Fig 1:**
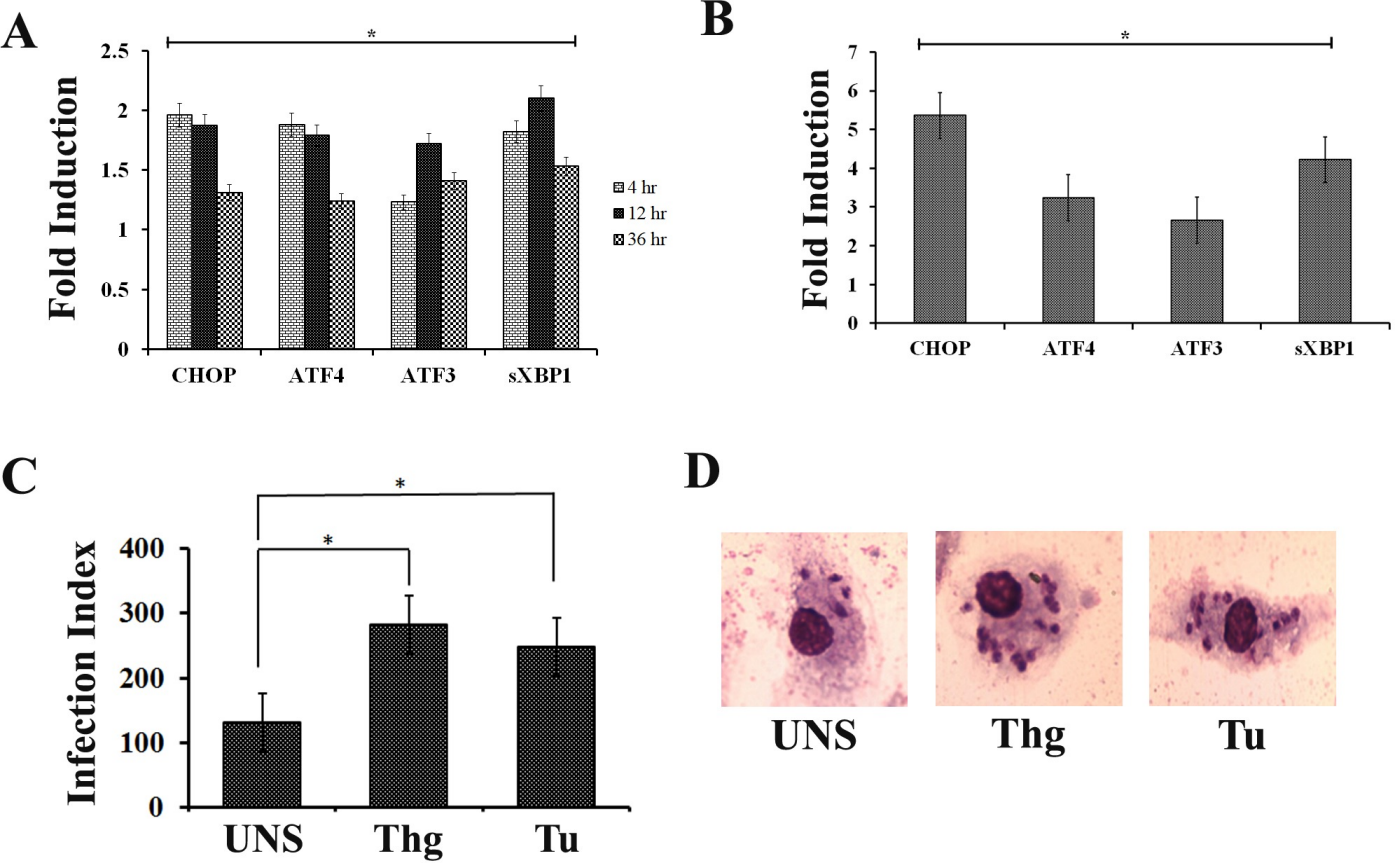
*L*.*donovani* infection induces UPR in RAW macrophages. **(A**) Gene expression profiling in RAW 267.4 macrophages 4 h, 12 h and 36 h after infection with *L*. *donovani* promastigotes. The graph shows the fold changes in comparison to the control (non-infected). (**B**) Gene expression in RAW 267.4 macrophages following thapsigargin (1.0μM/ 1hr) exposure. The graph shows the fold changes in comparison to the control (DMSO) values. (**C**) The induction of ER stress by thapsigargin (Thg) or tunicamycin (Tu) favours *L*. *donovani* infection. RAW264.7 cells pre-treated with thapsigargin for 1 h were infected with *L*. *donovani* promastigotes (10:1 MOI). After 36 h of infection, the cells were fixed with methanol and stained with Giemsa, and the infection index was calculated (% *L*. *donovani* infected cells X number of amastigotes per cell). The graph represents the infection index at 36 h. (**D**) Images showing thapsigargin (Thg) and tunicamycin (Tu) pre-treated RAW macrophages subjected to *L*.*donovani* infection. Untreated RAW macrophages (UNS) were taken as control. Data are represented as the mean ± SEM of at least three experiments. (* p < 0.05).

*Leishmania* infection is known to elicit ER stress and activates UPR response in host. [16]. To ensure the same, expression level of ER stress marker genes viz; CHOP, ATF3, ATF4 and sXBP1 was monitored by qPCR at 4hr, 12hr and 36hr post infection. A significant increase in the expression of ER stress marker gene was observed after infection. At 4hr and 12hr post infection, the increase in level of marker genes was more prominent i.e. 1.5–2.0 folds higher (Fig 1A). At later stage of infection (36hr), a general decrease in all ER stress marker genes was observed however the value was higher compared to uninfected RAW cells. As a positive control, RAW cells treated with an ER stress inducer (thapsigargin 1.0 μM/ 1hr) were considered. The selected ER stress marker genes were found to be significantly upregulated with higher magnitude of induction (Fig 1B). GAPDH was chosen as reference gene since its expression did not change significantly following infection.

### ER stress inducers enhances *L*. *donovani* infection

As *Leishmania* infection induces ER stress in host macrophages, we further investigated the effect of generated ER stress on infection rate. RAW macrophages treated with thapsigargin (Thg) or tunicamycin (Tu) for 1 hour were infected with *L*. *donovani*. After 36 hours, the infection index showed significantly higher parasite burden in thapsigargin (Thg) or tunicamycin (Tu) treated cells compared with that in non-treated cells (UNS) (Fig 1C). The result suggests that ER stress induced RAW macrophages have enhanced parasite infection.

### *L. donovani* induces host PERK phosphorylation

**Fig 2:**
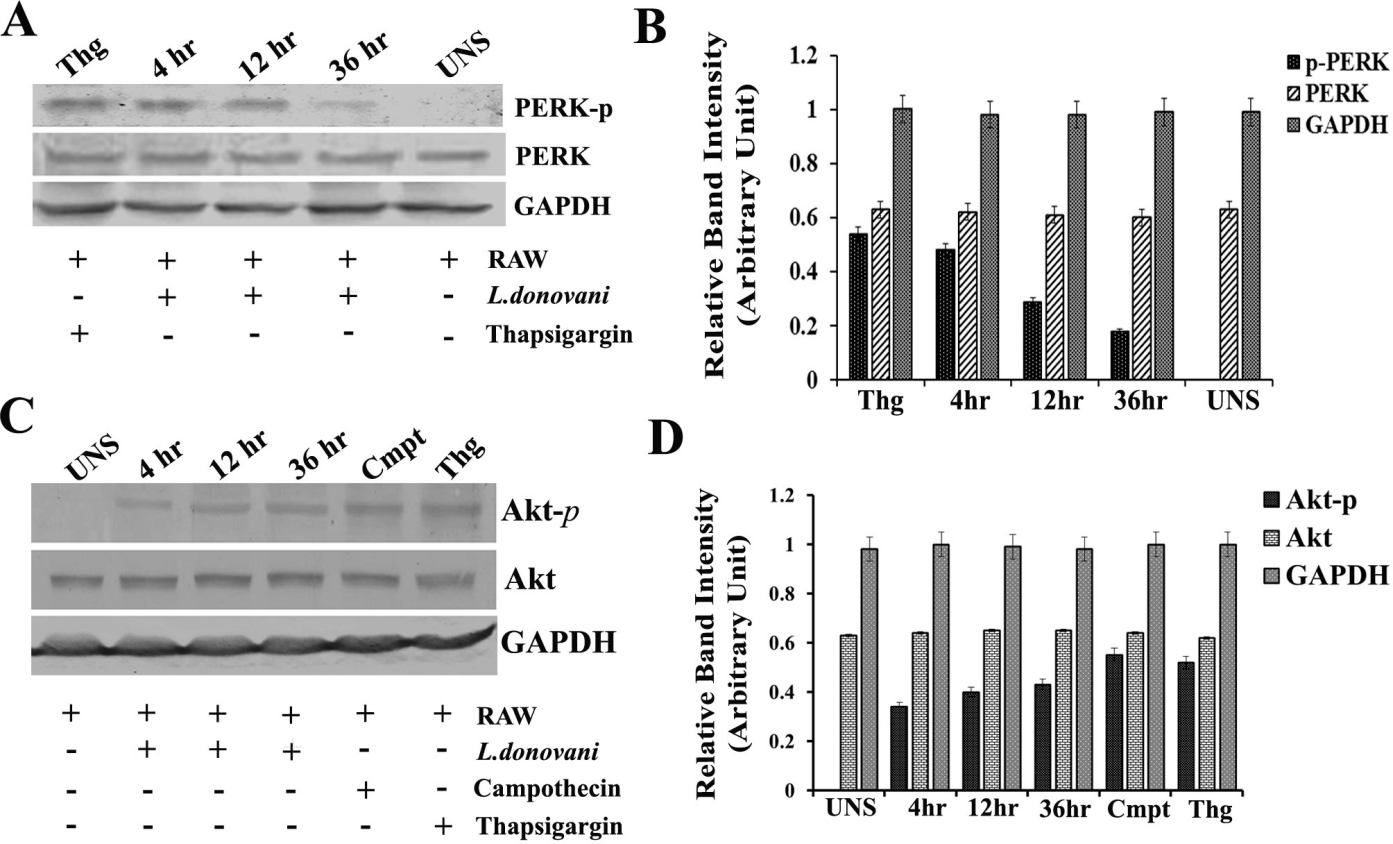
*L*.*donovani* infection induces host PERK and Akt phosphorylation. RAW 264.7 macrophages were infected with *L*. *donovani* promastigotes (10:1 MOI) and at 4 h, 12 h and 36 h post infection, harvested cells were lysed and equal amounts (50μg) were resolved by SDS-PAGE and analysed by Western blotting. (**A**) Western blot bands showing PERK phosphorylation at 4 h, 12 h and 36 h post infection when probed with phospho PERK antibodies (**B**) Densitomertic analysis of the blots for PERK phosphorylation. (**C**) Western blot bands for Akt phosphorylation probed with phospho-Akt showing Akt phosphorylation at 4 h, 12 h and 36 h post infection. (**D**) The relative density of the phospho- Akt bands from the blots was obtained and plotted during densitometry. Thg and Cmpt represent thapsigargin and camphothecin treatment respectively. Untreated or uninfected RAW macrophages (UNS) were taken as control. Data are represented as the mean ± SEM of at least three experiments.

Phosphorylation and simultaneous activation of PERK under stress is a major UPR which influences cellular adaptation and survival under stress through multiple mechanisms [18]. As the *L*.*donovani* infection induces ER stress in host macrophages, we further investigated the status and involvement of the host PERK in mounting UPR. Western blotting using phospho anti-PERK antibody revealed that in infected RAW cells, host PERK was phosphorylated (Fig 2A). At early stages of infection (4hr & 12hr) the degree of phosphorylation was higher compared to 36 hours post infection as shown in densitometry (Fig 2B). In case of thapsigargin treatment the phosphorylated status of PERK was confirmed and was taken as positive control for the experiment. No any phosphorylation was detected in untreated RAW macrophages.

### *L. donovani* infection induces Akt phosphorylation with delayed apoptosis of RAW cells

**Fig 3:**
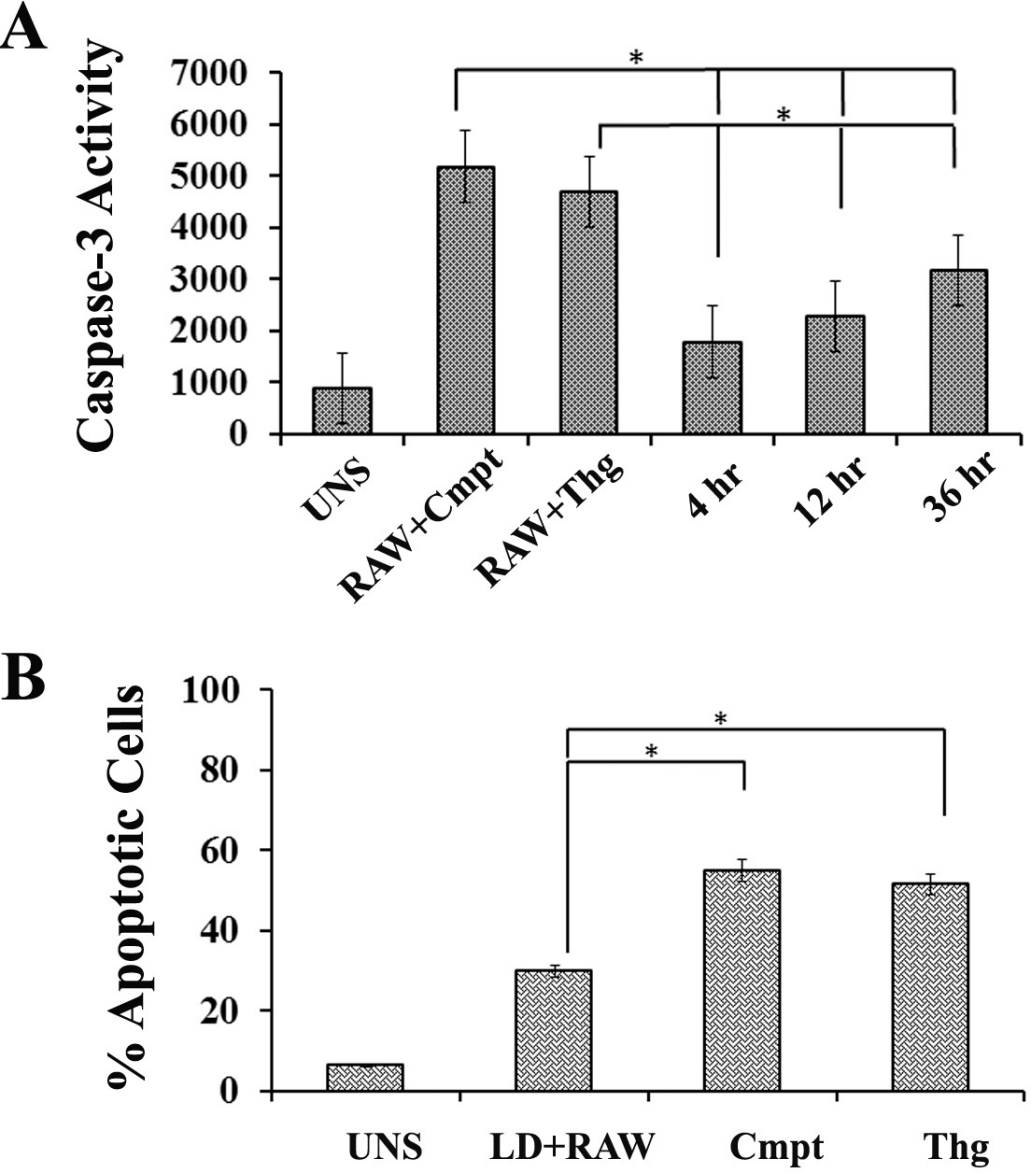
*L*. *donovani* infection reduces csapase-3 activity leading to decreased apoptotic induction in RAW macrophages. **A)** Caspase-3 activity in infected host macrophages: RAW267.4 macrophages were infected with *L*.*donovani* promastigotes. After 4 h, 12 h or 36 h of infection, infected cells were subjected to camphothecin (Cmpt) or thapsigargin (Thg) treatment for apoptotic induction and caspase-3 activity was determined. **B**) Apoptotic induction in host macrophages was determined after PI staining and analysed with flow cytometry under above mentioned conditions. The obtained values were plotted in terms of percent apoptotic cells. Uninfected RAW (UNS) macrophages in both the conditions were taken as control. Thg and Cmpt represent thapsigargin and camphothecin treatment respectively Data are represented as the mean ± SEM of at least three experiments. (* p < 0.05).

*Leishmania* parasites modulate the host cell by activating PI3K/Akt signalling and inhibiting caspase-3 activity which delays host cell apoptotic process. To confer this delayed apoptosis, Akt phosphorylation status, caspase3 activity and percentage of cells undergoing apoptosis was determine in RAW cells infected with *L*.*donovani*. A significant rise in Akt phosphorylation was observed in infected RAW cells compared to uninfected controls. The figure (Fig 2C) shows phosphorylation of Akt at early stages of infection (4hr &12hr) and was well maintained at 36 hours post infection. The infection had no effect on native Akt levels as represented in densitometric analysis of western blot bands (Fig 2D). Further, caspase-3 activity was accessed in uninfected or infected RAW cells or infected RAW macrophages treated with campothecin (2mM) or thapsigargin as inducers of apoptosis. The level of caspase-3 activity was found significantly high in uninfected macrophages subjected to campothecin (RAW+Cmpt) or thapsigargin (RAW+Thg) exposure than untreated control. In contrary, there was lower/limited activity of caspase-3 in macrophages that were first infected for 4hr, 12hr or 36hr with *L*. *donovani* promastigotes before exposure to campothecin or thapsigargin (Fig 3A). However, at 36hr post infection, the level of caspase-3 activity was higher than early stages of infection. Simultaneously, percentage of macrophages undergoing apoptosis was also determined in case of infected RAW macrophages compared to uninfected ones under exposure to apoptotic inducers. In uninfected RAW macrophages, the percentage of cells undergoing apoptosis was recorded significantly high compared to infected RAW cells (Fig 3B). Hence, the results were in agreement with previous findings that *Leishmania* infected macrophages are more tolerant to apoptotic induction [17].

### ER stress induces host IAP expression

**Fig 4:**
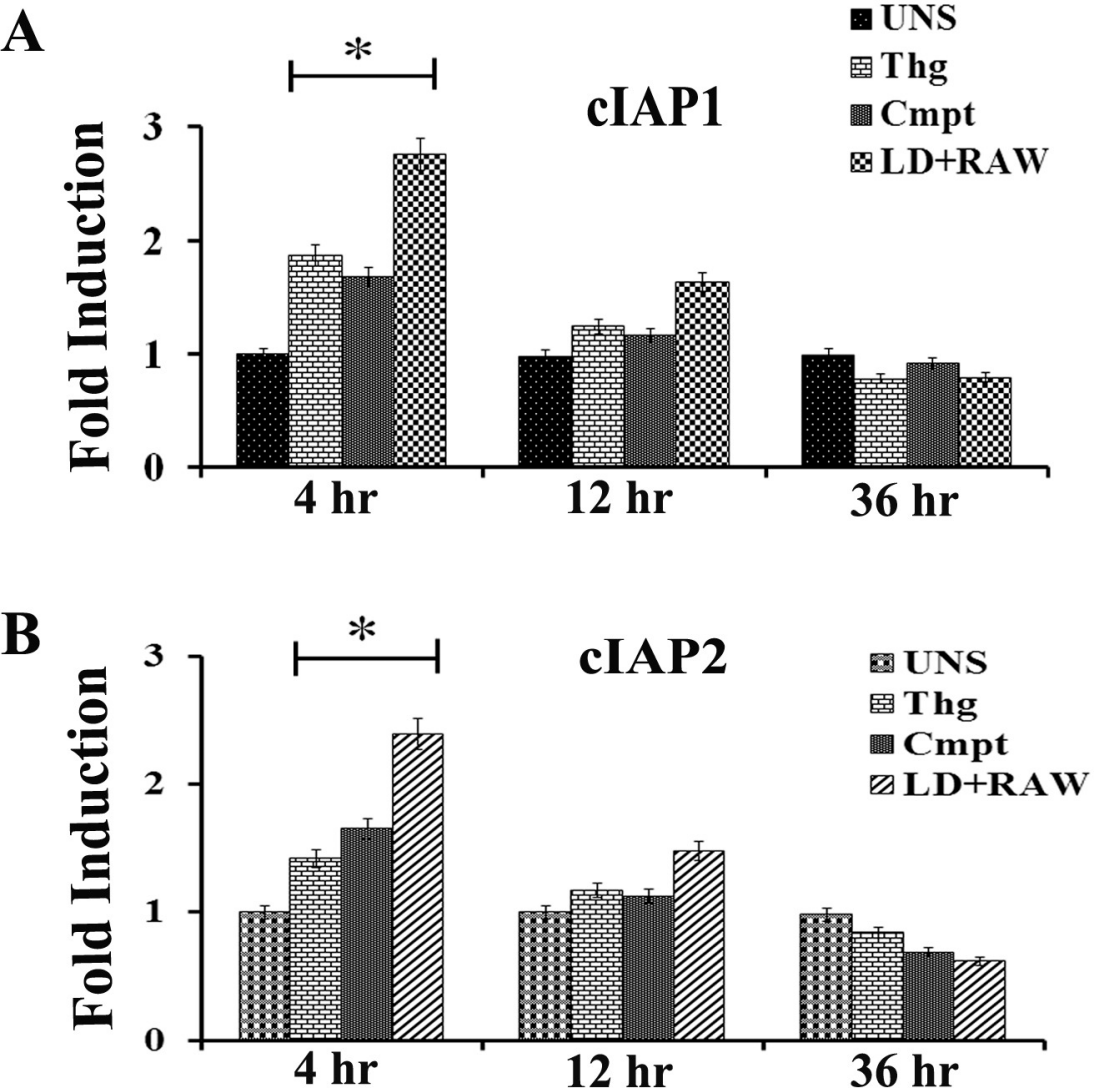
*L*. *donovani* infection induces transcription of host cIAP mRNA expression. **A**) Expression level of cIAP1 mRNA in RAW 267.4 macrophages infected with *L*. *donovani* for 4h, 12 h and 36 h or exposed to thapsigargin (Thg) or campothecin (Cmpt). **B**) Expression level of cIAP2 mRNA in RAW 267.4 macrophages infected with *L*. *donovani* for 4h, 12 h and 36 h or exposed to thapsigargin (Thg) or campothecin (Cmpt). Uninfected RAW 267.4 macrophages (UNS) without any treatment were taken as control. Data are represented as the mean ± SEM of at least three experiments. (* p < 0.05).

The mammalian inhibitor of apoptosis (IAP) gene family, particularly cellular IAPs (cIAP1 and cIAP2), provides cell protection during infection and against a variety of apoptotic stimuli [29, 30, 31, 32, 33]. It has been demonstrated that ER stress also induces the expression of cIAP1 and cIAP2 [34, 35] in a PERK-dependent manner [23]. As *L*.*donovani* infection delays host apoptosis induction, we accessed the level of cIAP1 and cIAP2 in infected RAW macrophages. Real time data revealed that the expression of cIAP1 and cIAP2 was induced in case of infected RAW cell, whereas no such induction was detected in uninfected macrophages. Induction of both cIAP1 and cIAP2 was recorded transiently highest at 4 hours post infection which gradually declined at latter stages of infection. Similar up regulation of cIAP1 and cIAP2 was also observed at mRNA level in RAW macrophages subjected to thapsigargin (1h/ 1μM) or campothecin (6h/ 2mM) treated RAW cell and were taken as control (Fig 4A and 4B).

### Host PERK inhibition promotes apoptosis

The above results reveal that *L*.*donovani* infection induces ER stress, phosphorylates host Akt molecule and delays host macrophage apoptosis which facilitates infection. Also, induced ER stress phosphorylates host PERK and leads to accumulation of cIAP mRNA. So to further explore the connection between *L*.*donovani* infections induced ER stress and host PERK phosphorylation mediated UPR in modulating host cell apoptosis, inhibition studies was carried out. For inhibiting PERK mediated UPR response, RAW 267.4 macrophages were pre-treated with GSK2606414, a specific inhibitor of PERK phosphorylation [36, 37, 38, 39, 40]; followed by washing before any infection or exposure as it is also effective against *Leishmania* PERK [26]. However, GSK2606414 pre-treatment did not affect the parasite entrance as initially at 4 hours, no any significant difference in terms of parasite entrance was observed in the GSK2606414 pre-treated RAW macrophages than untreated/normal one (S2 Fig).

**Fig 5:**
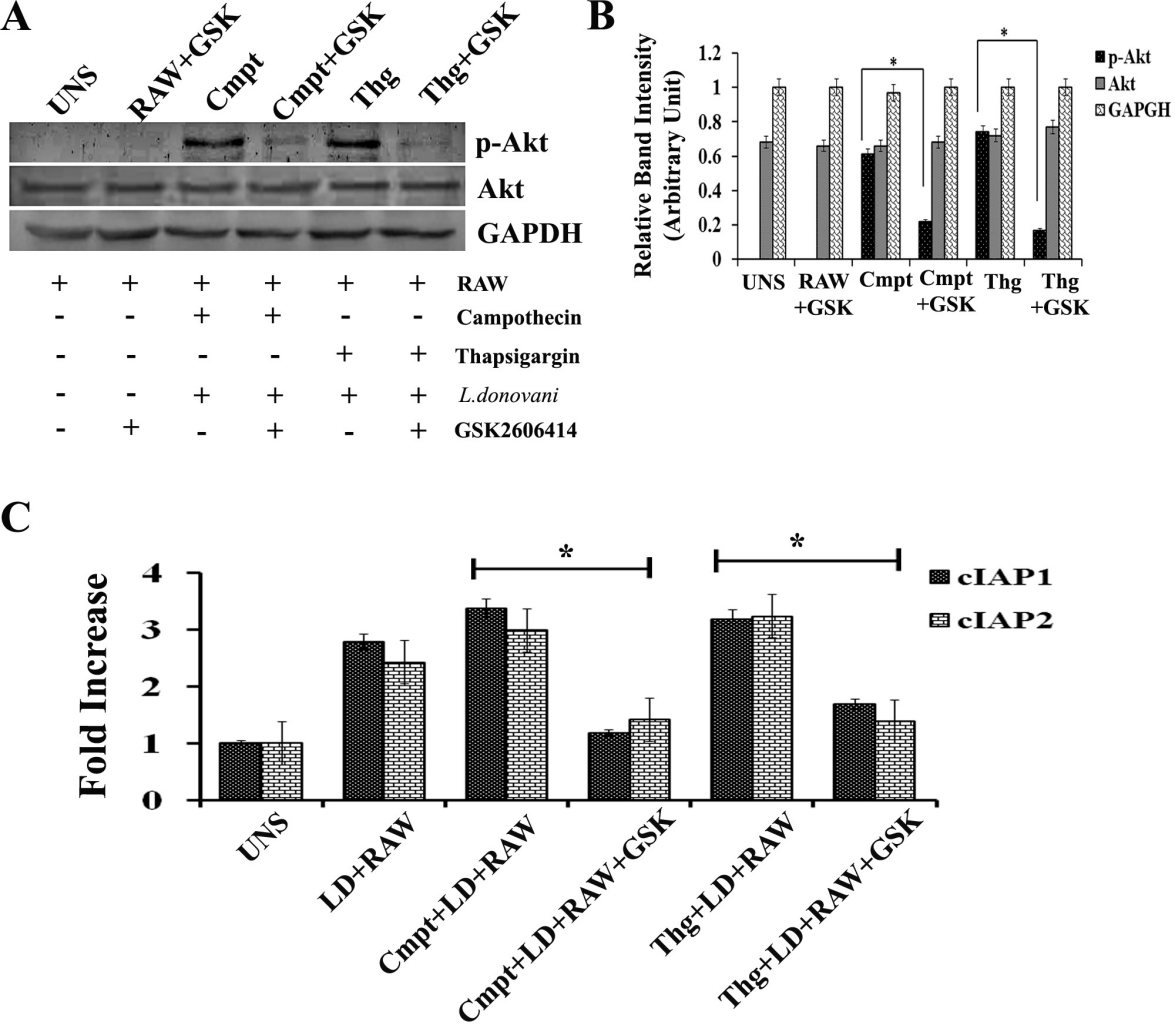
Inhibition of host PERK decreases Akt phosphorylation and inhibits transcription of host cIAP mRNA expression under exposure to apoptotic inducers. **(A**) RAW 267.4 macrophages pre-treated with GSK2606414 (2 hours) or untreated were infected with *L*.*donovani* promastigotes. After 4 h of infection, GSK pre-treated infected cells were exposed to campothecin (Cmpt+GSk) or thapsigargin (Thg+GSK). Similarly, untreated infected RAW macrophages were also exposed to campothecin (Cmpt) or thapsigargin (Thg). After treatment, cells were harvested and western analysis probed with phosphor-anti Akt, anti-Akt and GAPDH was performed from harvested cell lysates. (**B**) Band intensities of western blot bands after densitometry. (**C**) Relative expression of cIAP1 and cIAP2 mRNAs levels as analysed through real time in infected RAW 267.4 macrophages (4 h) either pre-treated with GSK2606414 or untreated, followed by campothecin (Cmpt+GSk) / (Cmpt) or thapsigargin (Thg+GSK) / (Thg) exposure. Infected RAW cell without any exposure were also taken in account. RAW 267.4 macrophages without any treatment or exposure were taken as control. Data are represented as the mean ± SEM of at least three experiments. (* p < 0.05).

**Fig 6:**
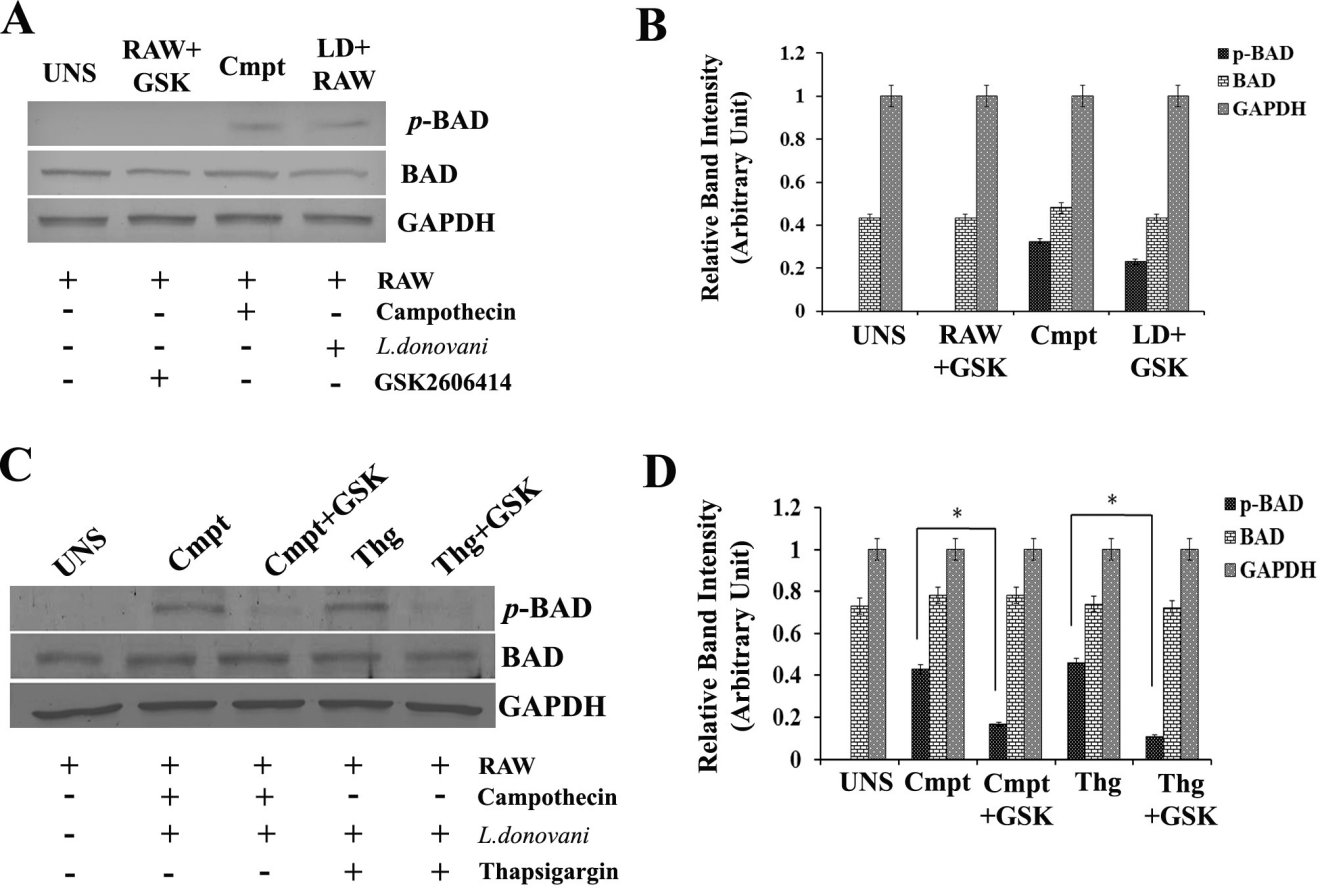
*L*.*donovani* infection phosphorylates BAD protein in a PERK dependent manner. **(A)** Western blot analysis to determine phosphorylated status of BAD protein using phosphos anti-BAD antibody in RAW 267.4 macrophages infected with *L*.*donovani* (LD) or exposed to campothecin (Cmpt). (**B**) Densitometry of western band for above experiment. **C**) Western analysis from lysates of infected RAW 267.4 macrophages pre-treated with GSK2606414 and exposed to campothecin (Cmpt+GSk) / thapsigargin (Thg+GSK) or untreated infected RAW cells subjected to campothecin (Cmpt) or thapsigargin (Thg) exposure. The band intensities were analysed densitometrically (**D**). For both the experiments, GAPDH was taken as control. Data are represented as the mean ± SEM of at least three experiments. (*p < 0.05).

**Fig 7:**
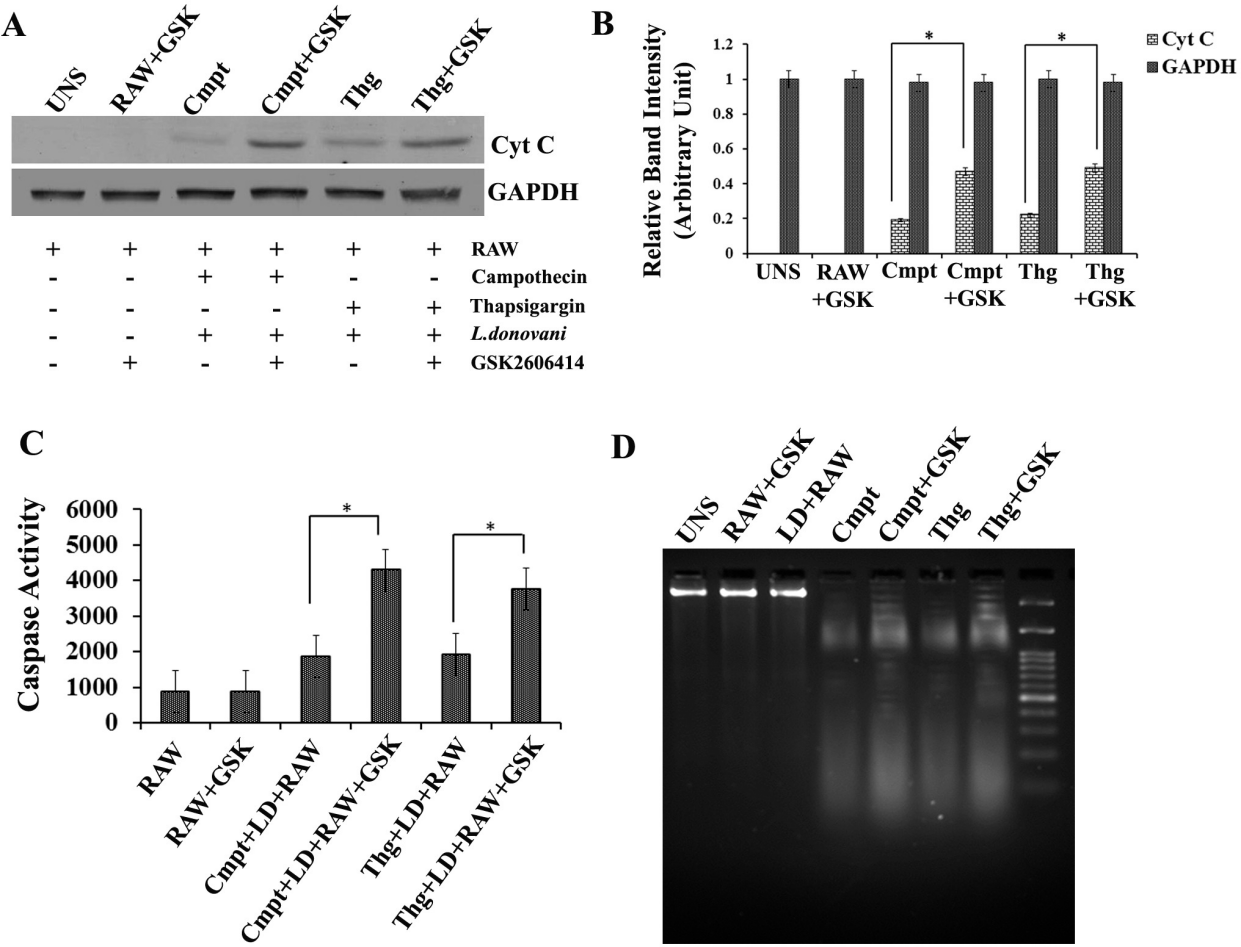
Host PERK inhibition leads to increased Cyt-C release and induces caspase-3 activity in infected RAW macrophages. RAW267.4 macrophages either pre-treated with GSK2606414 or untreated were infected with *L*.*donovani* promastigotes. After 4hr post infection, the infected cells were exposed to camphothecin (Cmpt+GSK/ Cmpt) or thapsigargin (Thg+GSK/ Thg) and the level of released Cyt C in cytosolic fraction was determined through western blot analysis using anti-Cyt C antibody. (**A**) Western blot result showing released Cyt C level in camphothecin (Cmpt) or thapsigargin (Thg) exposure or in GSK2606414 pre-treated RAW cell exposed to camphothecin (Cmpt+GSK) or thapsigargin (Thg+GSK). Untreated or uninfected RAW macrophages (UNS) or RAW cells treated with GSK2606414 (RAW+GSK) were taken as control. (**B**) Densitometry analysis of blot bands against Cyt C for above mentioned experimental conditions. **(C**) Caspase-3 activity in *L*.*donovani* infected RAW 267.4 macrophages after campothecin or thapsigargin treatment: Prior to infection, RAW macrophages were incubated with GSK2606414, washed and allowed for infection with L.donovani promastigotes. Infected RAW (4 h) were further exposed to campothecin (LD+RAW+Cmpt+GSK) or thapsigargin (LD+RAW+Thg+GSK). In another set, RAW macrophages without GSK2606414 treatment were allowed to infect followed by campothecin (LD+RAW+Cmpt) or thapsigargin (LD+RAW+Cmpt) treatment. Untreated or uninfected RAW macrophages (RAW) were taken as control. (**D**) DNA analysis by agarose gel electrophoresis revealed DNA fragmentation into oligonucleosome-sized fragments in control RAW 267.4 macrophages (UNS), RAW cell infected with *L*.*donovani* (RAW+LD), GSK pre-treated; infected RAW (4 h) exposed to campothecin (LD+RAW+Cmpt+GSK) or thapsigargin (LD+RAW+Thg+GSK). Untreated or uninfected RAW macrophages (UNS) or RAW cells treated with GSK2606414 (RAW+GSK) were taken as control. Data are represented as the mean ± SEM of at least three experiments (*p < 0.05).

**Fig 8:**
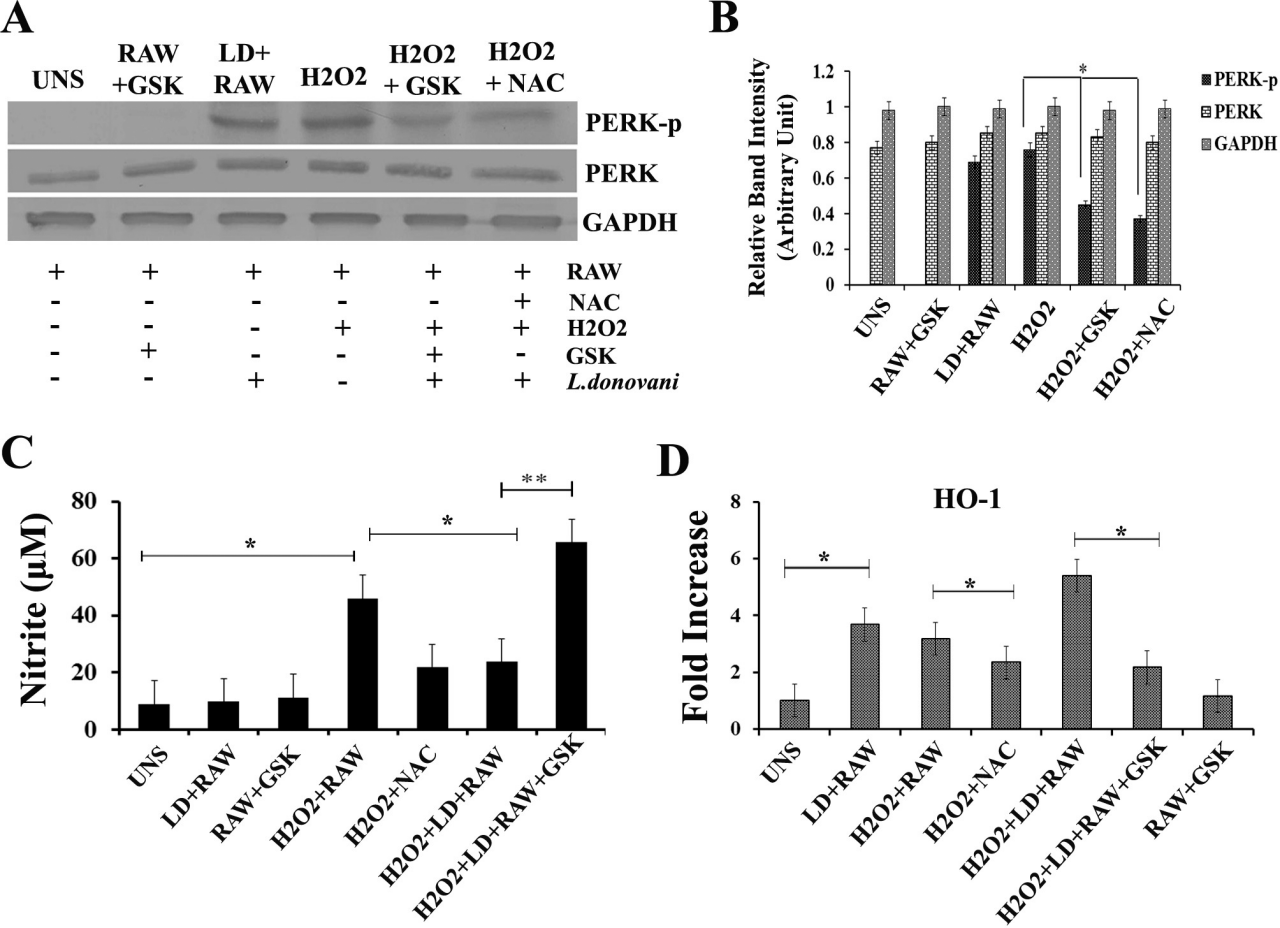
PERK phosphorylation in *L*.*donovani* infected RAW macrophages is required for protection against oxidative stress. **A)** Western blot analysis to determine host PERK phosphorylation status in *L*.*donovani* infected RAW 267.4 macrophages under oxidative exposure. GSK2606414 pre-treated or untreated RAW 267.4 macrophages, infected with *L*.*donovani* were exposed to H_2_O_2._ The cells were then harvested; lysed and equal amount of protein (50μg) was resolved on SDS-PAGE followed by probing with anti-phospho PERK antibody. Normal RAW macrophages (RAW) or *L*.*donovani* infected (LD+RAW) were considered as control. **B**) Densitometric analysis of western blot bands. **C**) Nitrite concentration was measured by Griess reaction in cell supernatant of infected RAW macrophages (LD+RAW) or in infected RAW cells exposed to H_2_O_2_ (LD+RAW+ H_2_O_2_) or pre-treated with GSK2606414 (LD+RAW+ H_2_O_2_+GSK) or in uninfected (H_2_O_2_+RAW) cells without any treatment. Untreated (UNS) or RAW macrophages exposed to H_2_O_2_ in presence of NAC (H_2_O_2_+ NAC) were taken as control. **D**) Real time plot to determine relative expression of HO-1 at mRNA level under above mentioned conditions.

- **The Akt mediated signalling pathway requires PERK phosphorylation**.
  An ER stress-inducing agent activates the PI3K–Akt signalling pathway [34, 41 and 42]. To determine whether PERK-dependent signals trigger Akt activation, RAW cells treated with campothecin or thapsigargin in presence or absence of GSK were harvested at the indicated intervals and Akt phosphorylation was determined by western analysis using antibodies specific for Akt phosphorylated at serine 473. Akt phosphorylation was observed in RAW cells exposed to campothecin or thapsigargin to alone, whereas significantly a low level of phosphorylated Akt was detected in GSK treated cells (RAW+GSK) (Fig 5A & 5B).
- **PERK inhibition regulate the expression of IAP mRNA**.
  Earlier work has revealed that PERK and Akt regulate the expression of IAP at transcriptional level [23]. Also in the above results we found that *L*.*donovani* infection induces cIAP1 and cIAP2 mRNA translation. Since IAP is directly related to anti-apoptotic mechanism, we determine the involvement of host PERK in regulation of cIAP expression under apoptotic stimulus. RAW macrophages untreated or pre-treated with GSK2606414 were allowed to infect by *L*.*donovani* promastigotes. After 4 hours post infection, infected cells were exposed to campothecin or thapsigargin for apoptotic induction. RNA was prepared and cDNA was reversed transcribed for further real time analysis of cIAP1 and cIAP2 mRNA levels. It was found that in GSK2606414 untreated cells, the level of cIAP1 and cIAP2 was significantly up-regulated by ~ 3.0 fold high compared to GSK pre-treated RAW macrophages (Fig 5C) under. So the result suggests that the mRNA expression of cIAP1 and cIAP2 is regulated in a PERK-dependent manner.
- ***L. donovani* infection phosphorylates BAD protein in a PERK dependent manner**.
  Phosphorylation of Akt may activate cascade of downstream molecules of the apoptotic pathway. To investigate the same, BAD protein phosphorylation status was monitored in *L*.*donovani* infected RAW macrophages. BAD protein is a member of proapoptotic Bcl-2 family which serves as an important regulator of apoptotic cell death [43]. Phosphorylation of BAD protein under various stimuli leads to its inactivation and divert cell fate against apoptosis. First we investigated the phosphorylation status of BAD in RAW macrophages under infected conditions. It was observed that the BAD protein was phosphorylated in RAW macrophages (Fig 6A) under exposure to campothecin. Further, the involvement of PERK in BAD phosphorylation was investigated in presence of GSK2606414. RAW macrophages either untreated or pre-treated with GSK2606414 were infected with *L*.*donovani* promastigotes. After 4 hours post infection, the macrophages were exposed to campothecin or thapsigargin and the phosphorylation of BAD protein in lysates was determined through western blot. In GSK2606414 treated RAW macrophages, the level of phosphorylated BAD was found to be significantly decreased than untreated infected RAW macrophages (Fig 6C & 6D). Hence the result summarises that PERK phosphorylation is required for BAD phosphorylation.
- **Inhibition of host PERK in infected RAW cells induces release of Mitochondrial Cytochrome C with increased caspase-3 activity**.
  Release of cytochrome C from mitochondria after disruption of outer mitochondrial membrane is an important marker indicating triggering of apoptosis in metazoans. RAW cell pre-treated with GSK or without it were infected with *L*.*donovani* promastigotes and in infected RAW macrophages (4 h post infection) released cytochrome C in cytoplasmic protein fraction was determined through western blotting using anti-Cyt C antibody after campothecin (2mM/6h) exposure. It was observed that the level of cytochrome C in GSK2606414 pre-treated infected RAW cells (Cmpt+GSK) was ~ 2.1 folds higher than in GSK2606414 untreated infected RAW cells exposed to campothecin (Cmpt) as revealed by densitometric analysis (Fig 7A & 7B). Similarly results were obtained in case of thapsigargin exposure. Normal RAW cells without any exposure or treatment were taken as control and did not show Cyt-C release. To further access the progression of apoptosis, caspase-3 activity was determined in cell lysates of infected RAW macrophages either pre-treated or untreated with GSK2606414 under campothecin or thapsigargin treatment. Compared to GSK2606414 untreated infected RAW cells, the level of caspase-3 activity was observed to be ~ 2.3 fold and ~ 2.0 fold higher in GSK pre-treated infected RAW cells under campothecin (Cmpt+LD+RAW+GSK) and thapsigargin (Thg+LD+RAW+GSK) exposure respectively (Fig 7C). Hence the results showed that the inhibition of host PERK phosphorylation leads to significant increased caspase-3 activity.
- **Inhibition of PERK in infected RAW cells results in increased DNA fragmentation**.
  One of the important identification features of apoptosis in mammalian cells is the fragmentation of cell DNA in multiples of 180–200 bp. Similarly we also measure the DNA fragmentation in *L*.*donovani* infected RAW267.4 in presence of GSK2606414 after exposure to apoptotic inducers. Agarose gel runs in Fig 7D shows that induction of apoptosis with campothecin or thapsigargin in *L*.*donovani* infected cells pre-treated with GSK2606414 (Cmpt+GSK or Thg+GSK) resulted in an increase in DNA fragmentation as compared to GSK2606414 untreated infected RAW cells (Cmpt or Thg). Control untreated macrophages (UNS) or L.donovani infected RAW cells (LD+RAW) did not show any DNA fragmentation. The DNA fragmentation assay revealed that inhibition of infection induced PERK phosphorylation resulted in an increased DNA fragmentation upon apoptotic induction.
- **Oxidative stress induces PERK Phosphorylation in RAW macrophages**.
  PERK mediated integrated endoplasmic reticulum stress response (IERSR) has a crucial role in redox homeostasis of the cell [44, 45]. To investigate the effect of oxidative stress on PERK of RAW macrophages in co-relation with *L*.*donovani* infection, phosphorylation status of PERK was determined. Western blot analysis using phospho-PERK antibody against protein lysates of RAW macrophages exposed to H_2_O_2_ either untreated (H_2_O_2_) or pre-treated with GSK2606414 (H_2_O_2_+GSK) or incubated with NAC (H_2_O_2_+NAC) revealed that the PERK enzyme was phosphorylated in H_2_O_2_ exposed RAW cells (Fig 8A); however the level of phosphorylation found to be significantly decreased in GSK pre-treatment or NAC incubation as represented in densitometric analysis of western blot bands (Fig 8B). The result suggests that oxidative stress equally serves as potent inducer of PERK phosphorylation compared to *L*.*donovani* infection which was considered as positive control in the same study. Normal RAW macrophages without any treatment or incubated along with GSK2606414 were taken as negative control in which no any phosphorylation was detected.
- **PERK phosphorylation regulates HO-1 expression and protects *L*. *donovani-*infected macrophages from oxidative stress**.
  PERK phosphorylation plays a critical role in protection against oxidative stress by promoting expression of HO-1, a fundamental anti-oxidative enzyme of eukaryotic system. During Leishmania infection, host PERK-ATF4-NRF2 mediated pathway regulates HO-1 expression to maintain redox balance [28]. To determine the effect PERK inhibition on *L*.*donovani* infected host cells during oxidative exposure, GSK2606414 pre-treated or untreated infected RAW 267.4 macrophages (4hr) were exposed to H_2_O_2_ and nitrite production as an indirect measure of nitric oxide (NO) levels was determined in cell supernatants. It was observed that NO level in H_2_O_2_ exposed uninfected RAW cell (H_2_O_2_ +RAW) was ~ 5 folds higher compared to infected RAW macrophages (UNS) but was ~2 folds higher in case of *L*.*donovani* infected RAW (H_2_O_2_ +LD+RAW) macrophages (Fig 8C). However, the level of NO was maximum ~ 7.0 times higher in infected RAW cells pre-treated with GSK2606414 (H_2_O_2_+LD+RAW+GSK). Uninfected RAW macrophages without any treatment were taken as control (UNS). Leishmania infection promotes expression of heme oxygenase (HO-1), a key antioxidant enzyme of host which facilitates parasite survival during oxidative burst to maintain redox homeostasis of host cell [28]. We further determined the expression of HO-1in infected RAW267.4 under oxidative environment. GSK2606414 pre-treated or untreated RAW macrophages; infected with *L*. *donovani* promastigotes (4hr) were exposed to H_2_O_2._ Following exposure, total mRNA was isolated, cDNA was prepared and HO-1 expression was determined. It was observed that the level of HO-1 was ~ 3.2 folds higher compared in H_2_O_2_ exposed RAW (H_2_O_2_+RAW) macrophages than normal untreated RAW cells (UNS). However in infected RAW macrophages (H2O2+LD+RAW) the level of was ~ 5.4 folds higher which results in lower NO production (Fig 8D). In GSK2606414 pre-treated infected RAW macrophages, a significant decreased level of HO-1 was observed; also reflected in higher NO production (Fig 8C).These data demonstrate that PERK phosphorylation mediated signaling pathway has an important role in the anti-oxidative cell response *during L*.*donovani* infection.

### Inhibition of host PERK induces apoptosis in infected RAW macrophages and decreases infection rate

**Fig 9:**
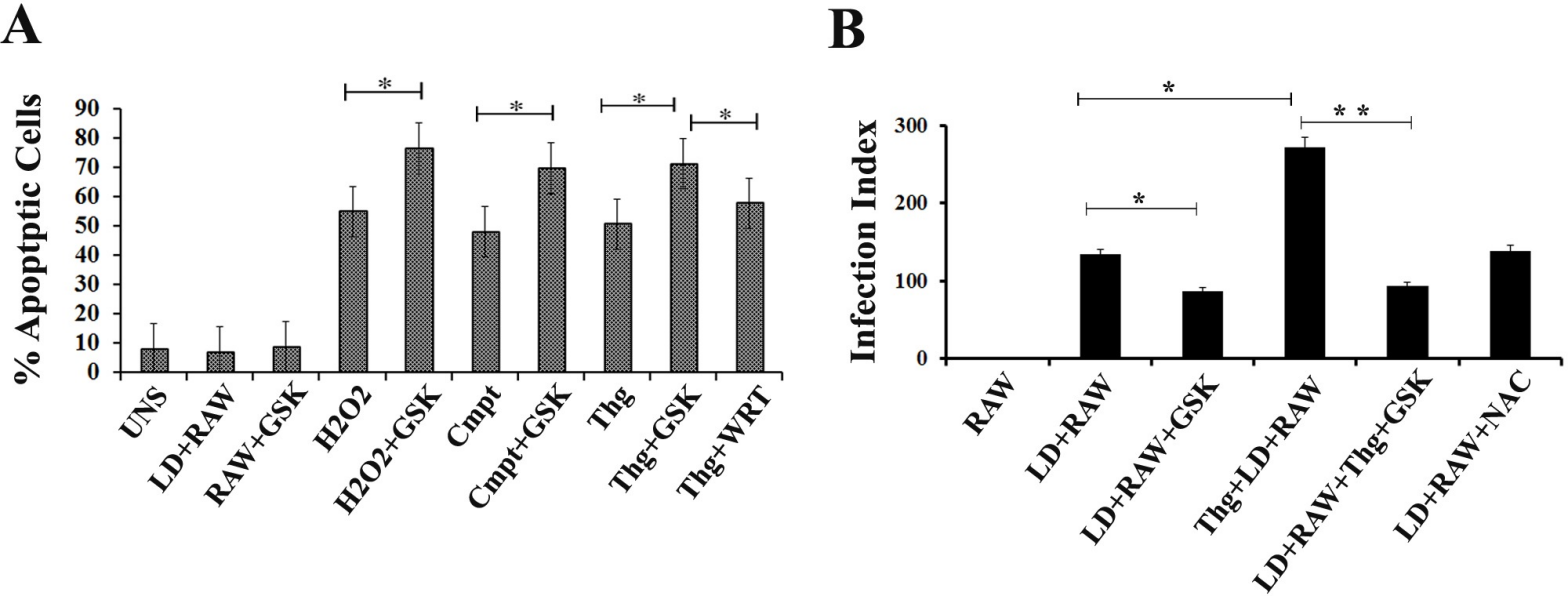
Inhibition of host PERK induces Apoptosis in infected RAW macrophages and decreases infection rate. (**A**) RAW macrophages undergoing apoptosis was determined using PI dye for infected RAW macrophages either treated with GSK2606414 subjected to H_2_O_2_ exposure (H_2_O_2_+RAW) or campothecin (Cmpt+RAW) or thapsigargin (Thg+RAW) and for untreated RAW macrophages exposed to H_2_O_2_ (H_2_O_2_)_,_ campothecin (Cmpt) and thapsigargin (Thg). Normal uninfected RAW macrophages without any treatment (UNS) or infected with *L*.*donovani* (LD+RAW) or incubated along with wortmanin subjected to thapsigargin exposure (Thg+WRT) were considered as control. **B)** RAW macrophages either thapsigargin exposed (Thg+LD+RAW) or NAC incubated (LD+RAW+NAC) were infected with *L*. *donovani* in 10:1 MOI. Also, GSK2606414 pre-treated RAW macrophages (LD+RAW+GSK) or followed by thapsigargin exposure (LD+RAW+Thg+GSK) were infected. After 36 h of infection, the cells were fixed with methanol and stained with Giemsa, and the infection index was calculated (percent *L*. *donovani* infected cells X number of amastigotes per cell) Uninfected RAW macrophages (RAW) or normal RAW macrophages infected with *L*.*donovani* (LD+RAW) were taken as control (Significant difference * (*P* < 0.05).

Finally, the percentage of infected RAW macrophages undergoing apoptosis after campothecin or thapsigargin or H_2_O_2_ exposure was determined in presence or absence of GSK2606414. The results revealed that GSK2606414 inhibition significantly increases the percentage of cells undergoing apoptosis (Fig 9A). Under H_2_O_2_ exposure, inhibition of PERK phosphorylation leads to significant increase in apoptotic cell percentage. *L*.*donovani* infected RAW macrophages in presence of Wortamanian also showed increased apoptotic rate; however the change was less significant compared to inhibitory effect of GSK2606414, suggesting that the effect of PERK inhibition is more prominent.

As the ER stress favours *L*.*donovani* infection in RAW macrophages, infection rate after inhibition of host PERK was investigated. RAW macrophages pre-treated with GSK2606414 were infected with *L*.*donovani* promastigotes. After 36 hours, infection status was determined in terms of infection index. In case of PERK inhibition, a drastic decrease in parasite burden was observed compared to untreated normal RAW macrophages subjected to infection as represented in infection index (Fig 9B). The similar pattern was also reported in thapsigargin induced RAW cells. This finding suggests that PERK mediated UPR is required for successful infection.

## Discussion

Various intracellular parasites deploy different strategies for their survival inside host; however one thing common among them is delayed/resistance against induced apoptosis via wide range of agents [46, 47]. Leishmaniasis disease is mainly contributed to the parasite ability to modulate host machinery to thrive the harsh phagolysosomal environment. *Leishmania* parasite deploy different strategies like; increases expression of host redox homeostasis enzymes like HO-1, SOD-1or catalase to sustain oxidative burst [10, 15], delays host cell apoptosis via PI3K-Akt pathway [17], induces ER stress to escape cellular defence, increases expression of IFN1-b etc.

In accordance with previous findings, we observed that *L*.*donovani* infection induces ER stress in RAW macrophages and consecutively activates UPR of the host as the level of marker genes like ATF3, ATF4, sXBP1and CHOP were found upregulated (Fig 1A). A similar trend of gene expression was observed in thapsigargin treated RAW macrophages (Fig 1B), which further validated the observation. The magnitude of UPR induction during infection was comparatively lower than thapsigargin treatment which gradually decreases at later time points; might be a requirement for spread and progression of infection after successful establishment. Moreover, a general down regulation of gene expression at later stages of infection has been reported in literature [48, 49].

Akt mediated signalling pathway has its unique importance in apoptosis induction. *Leishmania* parasite, as a survival strategy delays the apoptotic induction in host macrophages via phosphorylation and activation of Akt molecule [17]. ER stress also is capable of inducing Akt phosphorylation [23] which establishes a connection between UPR and PI3K-Akt pathway. In our studies, we observed Akt phosphorylation under apoptotic inducers exposure and *L*.*donovani* infection (Fig 2C). Simultaneously, with Akt phosphorylation, PERK was also found phosphorylated (Fig 2A) in infected macrophages. So the result bridges a connection between *Leishmania* infections, PERK mediated UPR activation and Akt pathway activation.

*Leishmania* infection confers host cell resistance to apoptosis by modulating PI3K/Akt signalling pathway [17] which we also confirmed in our studies using campothecin as apoptotic inducer. As compared to uninfected RAW macrophages, *Leishmania* infection brings about phosphorylation of host Akt with low caspase-3 activity in infected RAW cells. The level of caspase-3 activity was found higher in uninfected RAW cells which indicates early onset of apoptosis (Fig 3A). The mRNA level of cIAP1 and cIAP2 during *Leishmania* infection was comparatively higher than under apoptotic inducers (Fig 4) however; the induction was transient which decreases at later stages of infection. It has also been reported that at translational level both cIAP1 and cIAP2 protein continues to accumulate even at later stages and have key role in delayed apoptosis [23].

To further investigate the importance of PERK mediated UPR in *L*.*donovani* infected macrophages in connection with delayed apoptosis of host macrophages; inhibition studies using GSK2606414 was performed. RAW macrophages pre-treated with GSK2606414 were infected with *L*.*donovani* promastigotes. After 4 hours post infection, infected RAW macrophages were exposed to campothecin or thapsigargin for apoptotic induction and the molecular mechanism involved in apoptosis was investigated.

The level of Akt phosphorylation was found to be decreased in case of GSK treated macrophages compared to untreated one under thapsigargin exposure signifies that UPR-dependent Akt activation requires PERK phosphorylation (Fig 5A). Similar results were obtained in case of campothecin exposure; hence it can be concluded that induction of Akt phosphorylation under apoptotic stimulus is mediated via PERK phosphorylation.

Akt activation is implicated in the regulation of IAP gene transcription [34] and PERK regulates IAP expression in an Akt-dependent manner [23]. Similarly in GSK treated RAW macrophages, the level of cIAP1 and cIAP2 mRNAs was relatively in lower amount signifies that inhibition of host PERK hampers cIAP1 and cIAP2 accumulation (Fig 5C). Following the consequence, Akt phosphorylation brings about deactivation of downstream BAD protein. BAD being a proapoptotic molecule forwards the apoptosis process. Akt phosphorylation further phosphorylates BAD protein which checks its activity by facilitating its binding with 14.3.3 protein. In our findings, we observed decreased phosphorylation of BAD in GSK2606414 pre-treated infected macrophages than infected RAW macrophages without GSK2606414 (Fig 6C). Hence, PERK inhibition decreases infection induced BAD phosphorylation.

Phosphorylation of BAD render it inactive whereas, unphosphorylated BAD alters mitochondrial membrane permeability leading to cytochrome C leakage from inner mitochondrial space to cytosol which in turn activates proteases i.e. caspase like protein. Campothecin being an apoptotic activator enhances the release of cytochrome C in RAW macrophages. In *Leishmania* infected RAW macrophages, a low level of released cytochrome C suggests that infected macrophages are more tolerant to apoptotic inducers however, PERK inhibition leads to increased cytochrome C release even in infected RAW cell (Fig 7A).

For further determination of apoptotic progression, caspase-3 activity in lysates of infected RAW macrophages was accessed after campothecin or thapsigargin treatment. In our studies, we confirm the delayed onset of apoptosis in *L*.*donovani* infected RAW macrophages by interfering caspase-3 activity. However GSK2606414 treatment abolishes the effect of infection as higher caspase-3 activity was observed. In GSK pre-treated RAW macrophages, PERK inhibition alters the Akt mediated anti-apoptotic mechanism resulting higher caspase-3 activity (Fig 7C). Increased caspase-3 activity finally damage cell DNA to ensure apoptotic death of cell. DNA fragmentation analysis following campothecin treatment suggests that PERK inhibition leads to increased DNA damage even in infected macrophages (Fig 7D).

Apart from activation of caspase cascade, UPR induced Akt phosphorylation also regulates the level of cIAP mRNA in PERK dependent manner. In infected RAW cells, inhibition of PERK phosphorylation interferes with mRNA accumulation. A decreased cIAP1 and cIAP2 mRNA level due to initial inhibition of PERK; reflects the same controlling mechanism. Contrary to this, in *L*.*donovani* infected RAW cells, an upregulated level of cIAP1 and cIAP2 mRNA was observed (Fig 5C). Hence it can be extracted that parasite induces expression of IAP to delay apoptotic induction of host macrophages.

Increased level of released Cyt-C from mitochondria as a result of altered mitochondrial membrane potential; propagates the apoptosis [50]. ROS is one of the important reasons which alter mitochondrial membrane potential and leads to increased leakage of Cyt-C [51] responsible for apoptotic induction [52, 53]. Since PERK mediated signaling pathway has a distinct protective role against ROS; inhibition of the same resulted in increased NO level (Fig 8C). Also, inhibition of oxidative stress induced PERK phosphorylation leads to higher apoptotic cell percentage (Fig 9A) with less chances of survival of infected cells; hence decreased infection rate (Fig 9B).

**Fig 10:**
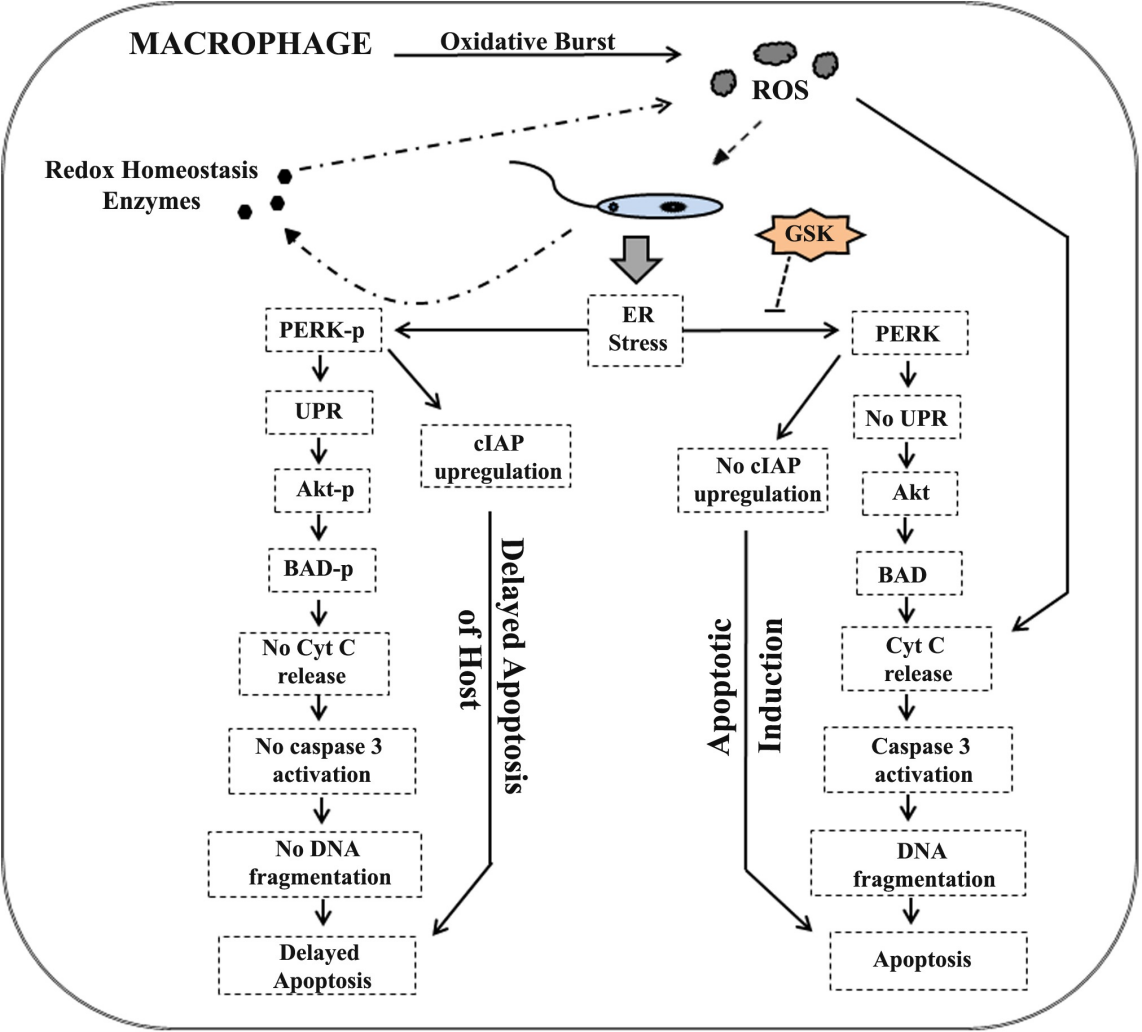
Leishmania infection induced UPR delays host macrophage apoptotic induction. During infection and entry of parasite inside host macrophages; parasite induces UPR in host which is mediated via phosphorylation of host PERK. PERK phosphorylation in turn activates the Akt pathway by inducing phosphorylation of Akt molecule, BAD protein phosphorylation and checks the release of mitochondrial cytochrome-C. Low level of released cytochrome-C fails to activate the caspase-3 enzymes as a result decreased DNA fragmentation, hence no/delayed apoptotic induction of host. Simultaneously, PERK phosphorylation also induces the transcription of anti-apoptotic class of proteins (cIAP1 & 2) which resist against apoptotic induction. However, inhibition of infection induced host PERK phosphorylation fails to activate this pathway and fates the host macrophage towards apoptosis. Also, PERK phosphorylation mediated UPR have a significant role in maintaining oxidative balance so parasite induced UPR might have a role in neutralizing host generated oxidative burst hence contributing to survival of Leishmania.

So overall it can be concluded that inhibition of host PERK during *L*.*donovani* infection, hampers the parasite ability to induce ER stress mediated host Akt phosphorylation and consecutively the phosphorylation of host BAD protein; which in turn alters mitochondrial membrane, increases cytochrome C release and finally DNA fragmentation. Simultaneously, decrease in IAP production in RAW macrophages due to PERK inhibition leads to an early onset of apoptotic process which ultimately results in decreased the parasitic burden (Fig 10).

Hence, findings from this study will help in better understanding of host-parasite interaction in relation with UPR activated PERK mediated mechanism in providing host cell resistance against apoptosis. It will open a new therapeutic option for drug target.

## Supporting information

S1 Fig Growth Curve of *L*.*donovani* promastigotes. Graph showing growth curve of *L*.*donovani* promastigotes in M199 media supplemented with 10% FBS. Initially, 10^5^ promastigotes per ml of culture was taken and grown for 8 days in BOD incubator. Cell viability was determined by trypan blue dye exclusion method at an interval of 24 hours.
(TIF)

S2 Fig Infection index at 4 hours. RAW macrophages either thapsigargin exposed (LD+RAW+Thg) or GSK2606414 pre-treated or normal/ untreated RAW macrophages (LD+RAW) were infected with L. donovani in 10:1 MOI. After 4 h of infection, the cells were fixed with methanol and stained with Giemsa, and the infection index was calculated (percent L. donovani infected cells X number of amastigotes per cell). Uninfected RAW macrophages (RAW) were taken as control. (Significant difference * (P < 0.05).
(TIF)
