# Supplementary figures and images for: Leishmania donovani induced Unfolded Protein Response delays host cell apoptosis in PERK dependent manner
Source: PLoS Negl Trop Dis. 2018 Jul 23;12(7):e0006646. doi: 10.1371/journal.pntd.0006646 (PMC6081962; doi:10.1371/journal.pntd.0006646)

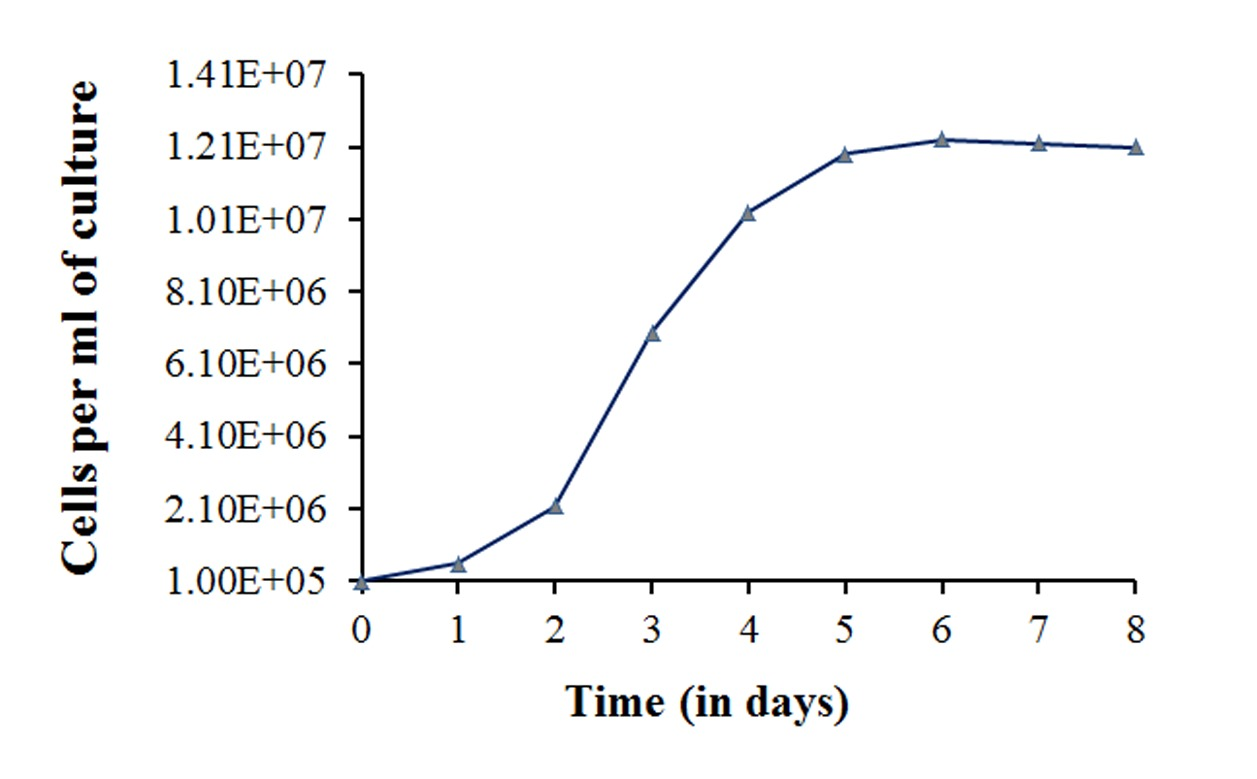

Supplement: S1 Fig — Graph showing growth curve of L.donovani promastigotes in M199 media supplemented with 10% FBS. Initially, 105 promastigotes per ml of culture was taken and grown for 8 days in BOD incubator. Cell viability was determined by trypan blue dye exclusion method at an interval of 24 hours. (TIF) [file pntd.0006646.s001.tif]

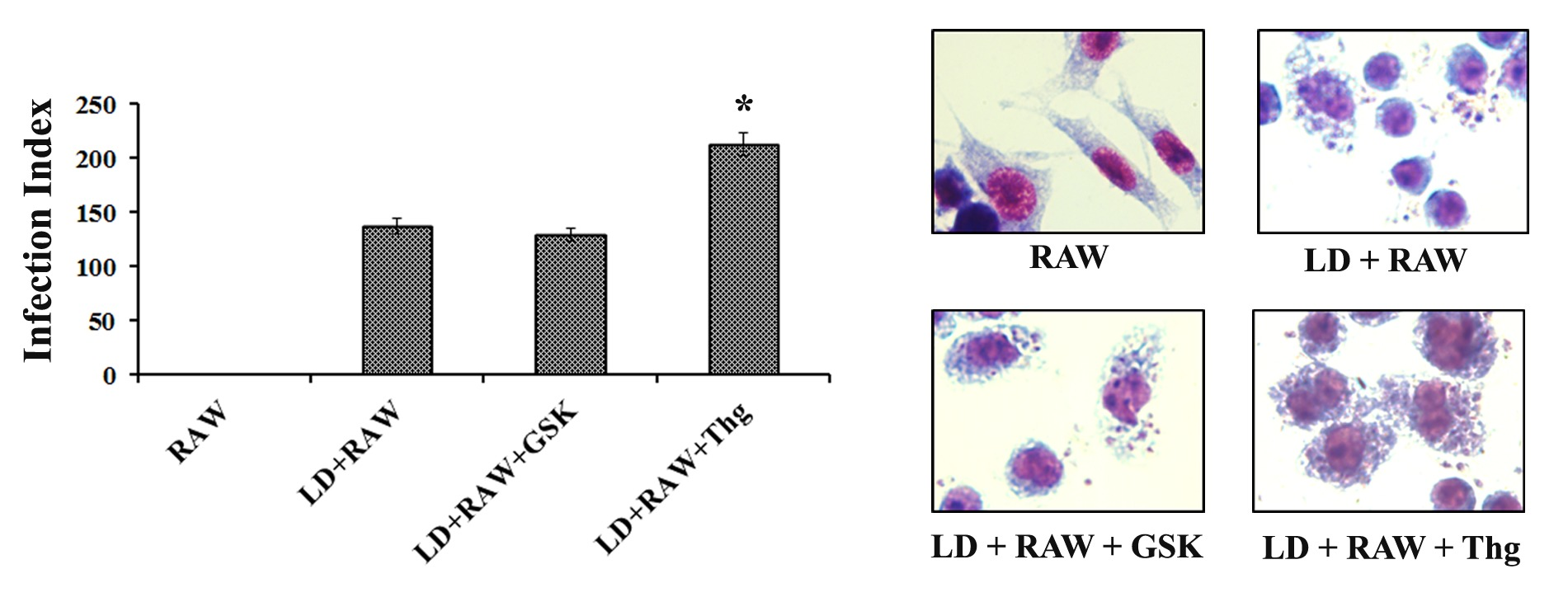

Supplement: S2 Fig — RAW macrophages either thapsigargin exposed (LD+RAW+Thg) or GSK2606414 pre-treated or normal/ untreated RAW macrophages (LD+RAW) were infected with L. donovani in 10:1 MOI. After 4 h of infection, the cells were fixed with methanol and stained with Giemsa, and the infection index was calculated (percent L. donovani infected cells X number of amastigotes per cell). Uninfected RAW macrophages (RAW) were taken as control. (Significant difference * (P < 0.05). (TIF) [file pntd.0006646.s002.tif]
